# Reducing the incidence of predictors of cardio-metabolic disease and dysglycaemia with lifestyle modification in at-risk persons – results of further analyses of DIABRISK-SL in those below 18 years of age

**DOI:** 10.1186/s12916-019-1398-2

**Published:** 2019-09-19

**Authors:** Nikolaos Fountoulakis, Mahen Wijesuriya, Luigi Gnudi, Martin Gulliford, Janaka Karalliedde

**Affiliations:** 1 School of Cardiovascular Medicine & Sciences, King’s College London, London, UK; 2 School of Population Health and Environmental Sciences, King’s College London, London, UK; 3 Diabetes Association of Sri Lanka, Rajagiriya, Sri Lanka

**Keywords:** Lifestyle modification, Diabetes prevention, South Asian, Cardio-metabolic risk, Randomised controlled trial

## Abstract

**Background:**

We have previously demonstrated in the DIABRISK-SL trial that a trimonthly pragmatic lifestyle modification (P-LSM), as compared to a 12-monthly LSM advice (C-LSM), significantly reduced the primary composite endpoint of predictors of cardio-metabolic disease (new onset type 2 diabetes (T2DM), hypertension, impaired glucose tolerance (IGT), impaired fasting glycaemia and markers of cardio-renal disease) in urban participants aged below 40 years with risk factors for T2DM.

**Main text:**

We now report results of post hoc analyses for those aged below 18 (*n* = 1725) in three age groups, specifically of 6–10 years (P-LSM *n* = 77, C-LSM *n* = 59), 10–14 years (P-LSM *n* = 534, C-LSM *n* = 556) and 14–18 years (P-LSM *n* = 239, C-LSM *n* = 260). There was no effect of P-LSM on the primary endpoint in participants aged below 10 years. Participants aged 10–14 years in the P-LSM intervention as compared to C-LSM had a lower incidence of the primary combined endpoint (87 vs. 106 cases; incident rate ratio (IRR) = 0.85, 95% confidence intervals (CI) 0.72–1.01; *P* = 0.07), driven mainly by the lower incidence of new onset hypertension (24 vs. 37 cases; IRR = 0.67, 95% CI 0.49–0.91; *P* = 0.012). Participants aged 14–18 years in the P-LSM intervention had a lower incidence of the composite endpoint (36 vs. 54 cases; IRR = 0.73, 95% CI 0.57–0.94; *P* = 0.015) as well as a lower incidence of IGT (12 vs. 21 cases; IRR = 0.6, 95% CI 0.39–0.92; *P* = 0.02), new onset hypertension (6 vs. 15 cases; IRR = 0.43, 95% CI 0.25–0.76; *P* = 0.004), and new onset dysglycaemia (composite of new T2DM, IGT and impaired fasting glycaemia) (30 vs. 46 cases; IRR = 0.74, 95% CI 0.56–0.97; *P* = 0.03) compared to those assigned to the C-LSM intervention. Limitations of the analyses are the post hoc approach and the small number of events in each group. There were no differences in retention between the two groups.

**Conclusions:**

Our results suggest that, in young South Asians aged between 10 and 18 years at risk of T2DM, a pragmatic lifestyle modification programme may reduce the incidence of predictors of T2DM and hypertension. There is a need for further studies in younger populations to evaluate the impact and feasibility of interventions to reduce the burden of T2DM and associated cardio-metabolic risk.

Please see related article: https://bmcmedicine.biomedcentral.com/articles/10.1186/s12916-017-0905-6

**Electronic supplementary material:**

The online version of this article (10.1186/s12916-019-1398-2) contains supplementary material, which is available to authorized users.

## Background

The Prevention of Cardio-metabolic Disease with Lifestyle Modification in Young Urban Sri Lankans (DIABRISK-SL) was a randomised controlled clinical trial comparing a trimonthly pragmatic lifestyle modification (P-LSM) programme with a less-intensive 12-monthly lifestyle modification (C-LSM) programme on a primary composite endpoint of predictors of cardio-metabolic disease in young, urban healthy participants aged below 40 years with risk factors for T2DM [1]. The study demonstrated the significant impact of a P-LSM programme on reducing a primary composite endpoint of predictors of cardio-metabolic disease (new onset type 2 diabetes (T2DM), hypertension, impaired glucose tolerance (IGT), impaired fasting glycaemia (IFG) and markers of cardio-renal disease) [1].

We appreciate the interest that Gkioni et al. [2] have shown in our published study by highlighting the importance of age differentiation, retention and missing data as factors that need to be considered to understand the full potential of the benefits observed in our study. We have outlined below the detailed response to each of these factors and the results of further analyses are also reported.

## Main text

### Age differentiation

Gkioni et al. [2] highlight the importance of further subcategory age differentiation as a factor to consider when interpreting the results of the intervention. As detailed in our original publication [1], the results for those below the age of 18 were post hoc and thus exploratory, and establish the platform and rationale for further studies in this population.

In response to the comment from Gkioni et al. [2], we have now performed further post hoc analyses and report the results of the intervention in age differentiated (6–10 years: P-LSM *n* = 77, C-LSM *n* = 59; 10–14 years: P-LSM *n* = 534, C-LSM *n* = 556; and 14–18 years: P-LSM *n* = 239, C-LSM *n* = 260) groups for the 1725 participants (P-LSM *n* = 850, C-LSM *n* = 875) aged below 18 years on the primary cardio-metabolic composite endpoint, new onset dysglycaemia (T2DM, IGT, IFG) and selected individual components of the primary composite endpoint (Additional file 1: Table S1). Definitions of the endpoints were as previously described [1].

No effect of P-LSM on cardio-metabolic endpoints was observed in participants aged below 10 years. There was evidence of a lower incidence of the primary combined endpoint in participants in the 10–14 years of age subgroup assigned to the P-LSM group (87 vs. 106 cases; incident rate ratio (IRR) = 0.85, 95% confidence intervals (CI) 0.72–1.01; *P* = 0.07), driven mainly by the lower incidence of new onset hypertension in the P-LSM group (24 vs. 37 cases; IRR = 0.67, 95% CI 0.49–0.91; *P* = 0.012) compared to the C-LSM group. Participants in the 14–18 years of age subgroup assigned to the P-LSM intervention arm had a lower incidence of the composite endpoint (36 vs. 54 cases; IRR = 0.73, 95% CI 0.57–0.94; *P* = 0.015) as well as a lower incidence of IGT (12 vs. 21 cases; IRR = 0.6, 95% CI 0.39–0.92; *P* = 0.02), new onset hypertension (6 vs. 15 cases; IRR = 0.43, 95% CI 0.25–0.76; *P* = 0.004) and new onset dysglycaemia (30 vs. 46 cases; IRR = 0.74, 95% CI 0.56–0.97; *P* = 0.03) compared to those assigned to the C-LSM intervention arm.

These results demonstrate that, in young South Asians at risk of T2DM, a P-LSM programme may reduce the incidence of predictors of diabetes and hypertension in those aged 10–18 years. As the number of participants in each of these subcategories are modest and the number of endpoint events (primary composite and components of the composite endpoint) is low, the results of these post hoc results should be considered as exploratory and hypothesis generating.

Gkioni et al. [2] also raise the point that food choices and activity options may be limited in young children and that engagement of their primary carers is key. We agree with this comment and, as reported in our original publication, lifestyle modification advice and guidance (which was focussed on food choices and detailed exercise options) was also given to the parents of younger children (age < 16 years) [1].

### Retention and missing data

There was no impact of retention on the duration of the study. The group-specific median duration of follow-up (interquartile range) was 1316 (730–1509) days in P-LSM and 1361 (806–1504) days in C-LSM (*P* = 0.5), with no indication of differential retention between groups. The number of participants lost to follow-up was also similar between the two groups over the duration of the study (P-LSM vs. C-LSM = 110 vs. 114 (year 1), 197 vs. 171 (year 2), 214 vs. 219 (year 3).

The original intention of our study was to follow-up participants for at least 5 years; however, as the screening phase (23,298 participants were screened to identify those at risk for potential participation, if eligible, in the clinical trial) took longer than originally expected and there were restrictions on available resources and funding, the follow-up period was reduced.

In our original publication, we also reported the baseline features of randomised participants (24%) who were not eligible for analyses as they did not attend any visits to receive lifestyle advice and we had no follow-up data available on them. These participants had similar baseline features as compared to those who were eligible for analyses, but the lack of follow-up information is a significant limitation of our work, as acknowledged in our publication [1].

The primary composite cardio-metabolic endpoint included new onset T2DM, hypertension, IGT, IFG, cardiovascular disease and renal disease. The Poisson model was employed for the primary analyses as this is suitable when analysing longitudinal data [3, 4]. Poisson regression analyses were performed, with person–time as exposure, to estimate the IRR with P-LSM as compared to C-LSM. We did not use last observation carried forward or any imputation in the primary or secondary key endpoint analyses.

End of study values for selected clinical and biochemical variables (biomarkers), adjusted for baseline value, age and sex for participants above and below 18 years of age in the P-LSM and C-LSM groups, were reported in our original manuscript as tables in the additional information files [1]. For these biomarker endpoints, the last observation was carried forward for those participants with missing endpoint values who did not complete the full duration of the trial.

As we previously reported [1], at the end of the trial, there was some evidence of lower fasting plasma glucose and 2-h post-oral glucose tolerance test plasma glucose levels in the P-LSM group compared to the C-LSM group in participants aged above 18 years of age only. Other markers, such as blood pressure and anthropometric and lipid parameters, were not different between the two groups at the end of the study. In our opinion, the changes in glucose levels observed were of modest clinical significance and, as these were not primary endpoints, we do not believe further imputation analyses are warranted.

## Conclusion

As the first randomised controlled trial of its type, in our original manuscript, we openly acknowledged the limitations of our study, which were thoroughly addressed therein to the satisfaction of the reviewers and the journal editors.

The results of our exploratory analyses suggest that, in young South Asians aged 10–18 years at risk of cardio-metabolic disease, a P-LSM programme may reduce the incidence of predictors of T2DM and hypertension. These results highlight the urgent requirement for further lifestyle modification studies in younger populations to investigate the impact of the intervention(s) on reducing new onset T2DM and associated cardiovascular risk factors.

DIABRISK-SL is an important first step and useful guide to developing locally applicable translation interventions that can address the growing burden of cardio-metabolic disease risk in younger persons.

## Additional file

Additional file 1: **Table S1.** Effect of pragmatic lifestyle modification (P-LSM) as compared to control lifestyle modification (C-LSM) on the incidence of the primary cardio-metabolic composite endpoint and its selected individual components in 1725 participants below 18 years of age stratified by age groups (PPTX 46 kb)

## Data Availability

The datasets used and/or analysed during the current study are available from the corresponding author on reasonable request.

## Abbreviations

CI: Confidence interval
C-LSM: 12-monthly lifestyle modification
IFG: Impaired fasting glycaemia
IGT: Impaired glucose tolerance
IRR: Incident rate ratio
P-LSM: Pragmatic lifestyle modification
T2DM: type 2 diabetes mellitus
