# Supplementary material for: Reducing the incidence of predictors of cardio-metabolic disease and dysglycaemia with lifestyle modification in at-risk persons – results of further analyses of DIABRISK-SL in those below 18 years of age
Source: BMC Med. 2019 Sep 19;17:162. doi: 10.1186/s12916-019-1398-2 (PMC6751787; doi:10.1186/s12916-019-1398-2)
Supplement: Supplementary file 1 — Table S1. Effect of pragmatic lifestyle modification (P-LSM) as compared to control lifestyle modification (C-LSM) on the incidence of the primary cardio-metabolic composite endpoint and its selected individual components in 1725 participants below 18 years of age stratified by age groups (PPTX 46 kb) [file 12916_2019_1398_MOESM1_ESM.pptx]

## Slide 1
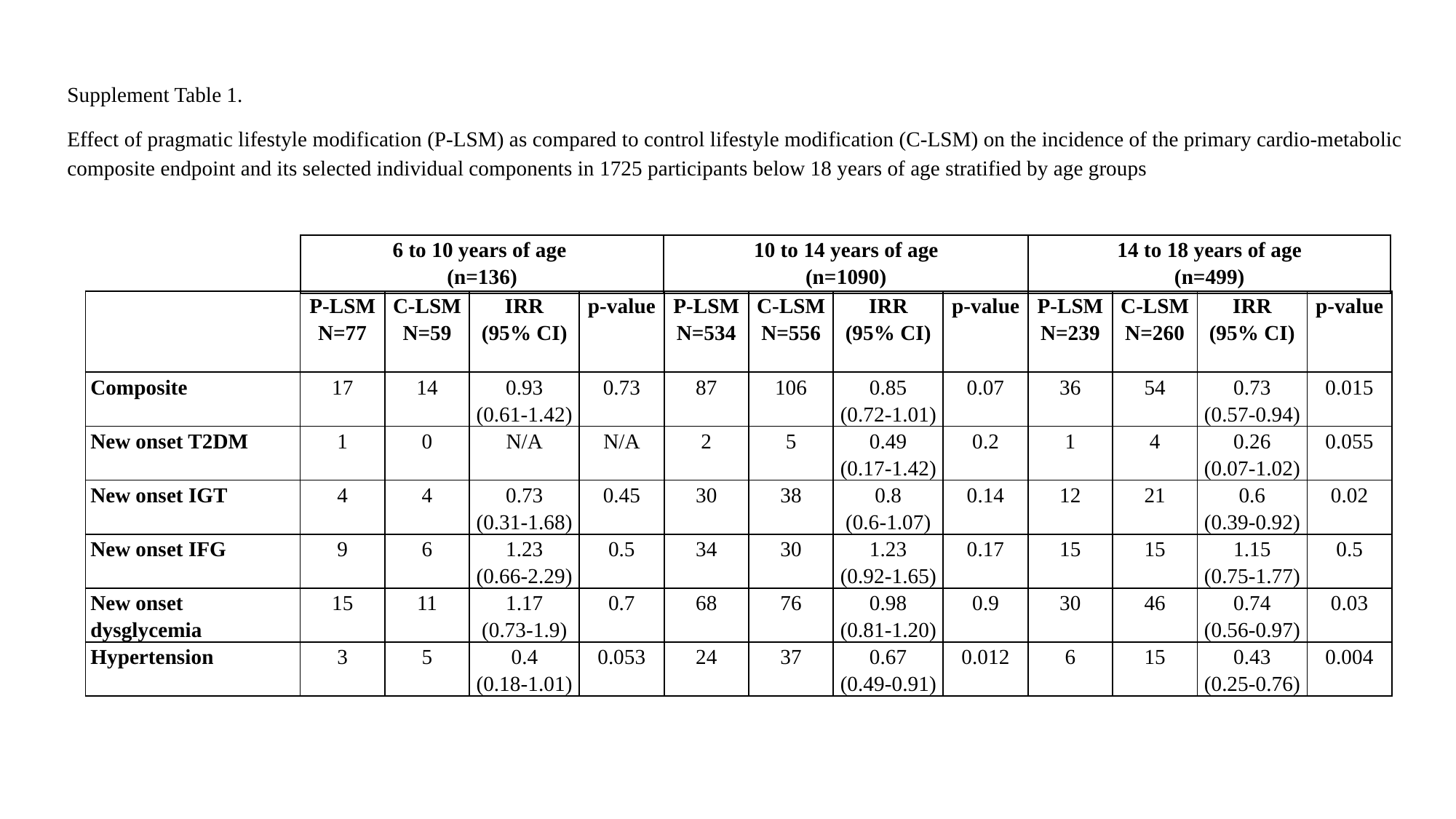

Supplement Table 1.
Effect of pragmatic lifestyle modification (P-LSM) as compared to control lifestyle modification (C-LSM) on the incidence of the primary cardio-metabolic composite endpoint and its selected individual components in 1725 participants below 18 years of age stratified by age groups
| 6 to 10 years of age (n=136) | 10 to 14 years of age (n=1090) | 14 to 18 years of age (n=499) |
| --- | --- | --- |
| | P-LSM N=77 | C-LSM N=59 | IRR (95% CI) | p-value | P-LSM N=534 | C-LSM N=556 | IRR (95% CI) | p-value | P-LSM N=239 | C-LSM N=260 | IRR (95% CI) | p-value |
| --- | --- | --- | --- | --- | --- | --- | --- | --- | --- | --- | --- | --- |
| Composite | 17 | 14 | 0.93 (0.61-1.42) | 0.73 | 87 | 106 | 0.85 (0.72-1.01) | 0.07 | 36 | 54 | 0.73 (0.57-0.94) | 0.015 |
| New onset T2DM | 1 | 0 | N/A | N/A | 2 | 5 | 0.49 (0.17-1.42) | 0.2 | 1 | 4 | 0.26 (0.07-1.02) | 0.055 |
| New onset IGT | 4 | 4 | 0.73 (0.31-1.68) | 0.45 | 30 | 38 | 0.8 (0.6-1.07) | 0.14 | 12 | 21 | 0.6 (0.39-0.92) | 0.02 |
| New onset IFG | 9 | 6 | 1.23 (0.66-2.29) | 0.5 | 34 | 30 | 1.23 (0.92-1.65) | 0.17 | 15 | 15 | 1.15 (0.75-1.77) | 0.5 |
| New onset dysglycemia | 15 | 11 | 1.17 (0.73-1.9) | 0.7 | 68 | 76 | 0.98 (0.81-1.20) | 0.9 | 30 | 46 | 0.74 (0.56-0.97) | 0.03 |
| Hypertension | 3 | 5 | 0.4 (0.18-1.01) | 0.053 | 24 | 37 | 0.67 (0.49-0.91) | 0.012 | 6 | 15 | 0.43 (0.25-0.76) | 0.004 |
